# Associations of a vegan diet with inflammatory biomarkers

**DOI:** 10.1038/s41598-020-58875-x

**Published:** 2020-02-06

**Authors:** Juliane Menzel, Ronald Biemann, Alessa Longree, Berend Isermann, Knut Mai, Matthias B. Schulze, Klaus Abraham, Cornelia Weikert

**Affiliations:** 1 German Federal Institute for Risk Assessment, Department of Food Safety, Berlin, Germany; 2 Institute for Clinical Chemistry and Pathobiochemistry, Otto-von-Guericke University Magdeburg, Magdeburg, Germany; 3 Institute of Laboratory Medicine, Clinical Chemistry and Molecular Diagnostics, University of Leipzig, Leipzig, Germany; 4 Department of Endocrinology & Metabolism, Charité - Universitätsmedizin Berlin, Berlin, Germany; 5 Center for Cardiovascular Research (CCR), Charité - Universitätsmedizin Berlin, Berlin, Germany; 6 Clinical Research Unit, Berlin Institute of Health, Berlin, Germany; 7 German Centre for Cardiovascular Research (DZHK), Partner Site Berlin, Berlin, Germany; 8 Department of Molecular Epidemiology, German Institute of Human Nutrition Potsdam–Rehbruecke, Nuthetal, Germany

**Keywords:** Endocrinology, Biomarkers, Risk factors, Biomarkers, Epidemiology

## Abstract

Vegetarian or vegan nutrition might influence inflammatory processes, thereby reducing the risk of chronic diseases. As the vegan diet becomes more importance in modern societies, data from the “Risks and Benefits of a Vegan Diet”-study has been used to investigate the associations of veganism with a comprehensive spectrum of inflammatory biomarkers, compared to omnivores. This cross-sectional study comprises 36 vegans and 36 omnivores (18 men and 18 women each) aged 30–60 years. No significant differences in any of the investigated inflammatory biomarkers (high-sensitivity C-reactive protein (hsCRP), interleukin-18 (IL-18), interleukin-1 receptor antagonist (IL-1 RA), intercellular adhesion molecule-1 (ICAM-1), adiponectin, omentin-1 and resistin) were observed between vegans and omnivores. However, the duration of a vegan diet was positively correlated with resistin (Spearman r = 0.59), IL-18 concentrations (Spearman r = 0.44) and IL-1 RA (Spearman r = 0.34). Moreover, the present study supports BMI and waist circumference as important factors influencing the inflammatory state. Further research is needed to evaluate associations between a vegan diet and inflammatory biomarkers to provide more evidence about the inflammatory state as underlying mechanisms of a vegan diet to influence the risk of numerous chronic diseases.

## Introduction

There is a growing trend for vegetarian and vegan diets in Germany and many Western countries^1^. These diets are typically higher in fruits, vegetables, whole-grains, legumes, nuts, and various soy products^2^, corresponding to larger amounts of antioxidant micronutrients such as vitamins C and E, phytochemicals and dietary fibre^2,3^. Especially, a growing trend toward veganism has arisen in the recent years, due to increasing awareness of the compassion for animals and environmental problems associated with livestock farming. Furthermore, more and more people are turning to a vegan diet also for the potential health benefits. Indeed, scientific evidence suggests that a vegan or vegetarian diet may be protective against many chronic diseases like type 2 diabetes^4^, cardiovascular diseases^5^, or cancer^6^. Recent research hypothesized links between low-grade inflammation and increased risk of various diseases, which are known to be triggered by underlying inflammation. Thus, inflammatory biomarkers may also act as intermediate risk factors in the development of chronic diseases. Actually, elevated levels of inflammatory markers like high-sensitivity C-reactive protein (hsCRP), or interleukin 18 (IL-18) are found to be associated with pathogenetic mechanisms of chronic diseases in type 2 diabetes^7^ or cardiovascular disease^8,9^. In contrast, concentrations of the anti-inflammatory hormone adiponectin were found to be inversely associated with these diseases^10,11^.

Recent evidence proposed that inflammatory biomarker profiles can be modulated by plant-based diets, showing an attenuation of inflammation markers as for instance CRP^12,13^ or soluble intercellular adhesion molecule 1 (sICAM-1)^12^. Similarly, meta-analyses noticed that vegetarian patterns were associated with lowered CRP concentrations^3,14^. Indeed, while the associations between vegetarian patterns on inflammatory biomarkers have been investigated in some studies, comprehensive scientific research on the impact of an exclusive vegan diet on inflammatory biomarkers is still missing. As the vegan diet becomes more importance in modern societies, the present “Risks and Benefits of a Vegan Diet” study (RBVD-Study) aimed to investigate the association between a vegan diet and inflammation by analyzing data of vegans and omnivores on a comprehensive spectrum of inflammatory biomarkers, i.e. high-sensitivity C-reactive protein (hsCRP), interleukin-18 (IL-18), interleukin-1 receptor antagonist (IL-1 RA), intercellular adhesion molecule-1 (ICAM-1), adiponectin, omentin-1 and resistin.

## Results

The distribution of general characteristics of the 72 sex- and age matched participants is shown in Table 1, according to vegan (n = 36) or omnivorous diet (n = 36). The median duration of veganism was 4.8 years (IQR: 3.1–8.7). As expected, we observed no differences in sex and age between vegans and omnivores (vegans: 50% male, median age: 37.5 years (min-max: 30.0–57.0); omnivores: 50% male, median age: 38.5 years (min-max: 30.0–57.0)). Moreover, we observed no differences in waist circumference, physical activity, smoking status, education status or alcohol consumption (all p > 0.05). However, vegans had a higher intake of vegetables, whereas no difference in fruit intake has been observed. Further, compared to omnivores, the plasma levels of saturated fatty acids (SFA) were lower in vegans, while levels of poly-unsaturated fatty acids (PUFA) were higher.

**Table 1:** Characteristics of the study population according to a vegan or omnivorous diet (n = 72).

	Vegans (n = 36)	Omnivores (n = 36)	*p-value*
Duration vegan diet [years]	4.8 (3.1–8.7)		
Men [%]	50% (18)	50% (18)	
Age [years]	37.5 (32.5–44.0)	38.5 (32.0–46.0)	*0.75*
Waist circumference [cm]			
Women	73.1 ± 6.9	77.2 ± 6.2	*0.07*
Men	84.5 ± 8.9	86.0 ± 6.1	*0.56*
Physical Activity [h/week]	2.8 (0.9–3.8)	2.3 (1.2–4.1)	*0.69*
Walking [h/week]	7.0 (5.0–12.0)	5.5 (3.5–11.8)	*0.15*
Smoking status [%]			*0.30*
Non-smoker	66.7% (24)	58.3% (21)	
Ex-Smoker	22.2% (8)	16.7% (6)	
Smoker	11.1% (4)	25.0% (9)	
Education [%]			*0.60*
Low	0.0% (0)	2.8% (1)	
Intermediate	30.6% (11)	30.6% (11)	
High	69.4% (25)	66.7% (24)	
Diet			
Alcohol consumption [g/d]			
Women	0.10 (0.00–4.69)	0.21 (0.02–4.88)	*0.22*
Men	0.04 (0.00–2.00)	3.85 (0.36–16.2)	*0.09*
Consumption fruits and vegetables [g/d]			
Fruits	185.5 (94.7–344.8)	152.3 (62.2–215.2)	*0.14*
Vegetables	459.5 (228.8–635.8)	226.8 (114.0–302.2)	*0.0001*
Total consumption	683.0 (463.0–887.8)	378.3 (216.0–523.0)	<*0.0001*
Plasma phospholipid fatty acids proportions [%]			
Total saturated fatty acids	46.0 (44.6–46.6)	47.3 (46.8–47.6)	<*0.0001*
Total mono-unsaturated fatty acids	12.8 (12.0–14.3)	12.3 (11.6–13.8)	*0.22*
Total poly-unsaturated fatty acids	41.0 (39.9–42.4)	39.9 (38.9–41.0)	*0.008*

Variables expressed as percentage (n) or mean ± SD or median (IQR).

As depicted in Table 2, we observed no significant differences of the inflammatory biomarkers i.e. adiponectin, ICAM-1, IL-18, IL-1 RA, omentin-1 or resistin between vegans in comparison to omnivores (all> 0.05). Regarding hsCRP in the unadjusted model, omnivores were with a tendency more likely to have higher hsCRP levels (0.94 mg/l (95%-CI 0.65–1.28)) compared to vegans (0.60 mg/l (95%-CI 0.36–0.87), p = 0.09), nevertheless, with additionally adjustment of lifestyle factors this difference was further diminished (p = 0.35, Table 2). Interestingly, the duration of a vegan diet was positively correlated with IL-18 (Spearman r = 0.44, p = 0.02, Table 3). Further, long-time vegans (>4.8 years) were with a tendency more likely to have lower hsCRP level (0.50 mg/l (95%-CI 0.21-0.85) compared to vegans adhering to a vegan diet less than 4.8 years (0.85 mg/l (95%-CI 0.46-1.33), p = 0.09). IL-1 RA was positively correlated with the duration of a vegan diet in model 1 (Spearman r = 0.35, p = 0.03), however, this association was diminished with additionally adjustment (Table 3). Moreover, increased resistin concentrations were highly correlated with the duration of a vegan diet (Spearman r = 0.59, p = 0.0008, model 2). Moreover, waist circumference and BMI were positively correlated with hsCRP, ICAM-1, IL-1 RA and inversely correlated with adiponectin and omentin-1, although the correlations were less pronounced for BMI (Table 3). As shown in Table 3, plasma concentrations of SFA were positively correlated to resistin, and inversely correlated to the anti-inflammatory biomarker adiponectin and omentin-1. Higher levels of PUFA were correlated with lower levels of hsCRP (Table 3). For consumption of fruits and vegetables we noticed no association to any of the investigated inflammatory biomarkers (Table 3). In sensitivity analyses, the observed associations were not substantially altered neither by the exclusion of participants taking anti-rheumatic or analgesic drugs, nor the exclusion of extreme concentrations of the inflammatory biomarkers or the additionally adjustment for type of diet (data not shown).

**Table 2:** Inflammatory biomarkers according to a vegan or omnivorous diet (n = 72).

	Vegans (n = 36)	Omnivores (n = 36)	*p-value*
Adiponectin [ng/ml]^a^			
Model 1	4.37 (3.84–4.96)	4.06 (3.57–4.62)	*0.43*
Model 2	4.44 (3.34–5.90)	4.15 (3.17–5.43)	*0.46*
hsCRP [mg/l]^a^			
Model 1	0.60 (0.36–0.87)	0.94 (0.65–1.28)	*0.09*
Model 2	0.44 (0.00–1.08)	0.61 (0.14–1.27)	*0.35*
ICAM-1 [ng/ml]^a^			
Model 1	531.3 (498.6–566.0)	557.5 (523.3 594.0)	*0.29*
Model 2	600.8 (523.9–689.1)	615.2 (540.3–700.5)	*0.60*
IL-18 [pg/ml]^a^			
Model 1	44.4 (29.6–66.4)	55.5 (37.1–82.8)	*0.44*
Model 2	72.6 (28.2–184.6)	95.6 (39.2–231.0)	*0.37*
IL-1 RA [pg/ml]^a^			
Model 1	203.0 (165.2–249.4)	199.7 (162.5–245.3)	*0.91*
Model 2	175.5 (107.5–286.5)	170.3 (107.0–270.8)	*0.85*
Omentin-1 [ng/ml]^b^			
Model 1	501.4 (449.6–553.3)	505.0 (453.1–556.9)	*0.92*
Model 2	511.7 (396.6–626.8)	507.7 (398.7–616.7)	*0.91*
Resistin [ng/ml]^b^			
Model 1	6.85 (6.22–7.47)	7.20 (6.58–7.82)	*0.43*
Model 2	5.87 (4.51–7.23)	6.38 (5.09–7.67)	*0.25*

^a^Expressed as geometric mean (95%-CI), ^b^Expressed as mean (95%-CI), Model 1: unadjusted, Model 2: adjusted for age, sex, smoking status, education, waist circumference, physical activity, alcohol consumption.

**Table 3:** Spearman correlations of inflammatory biomarkers with duration of a vegan diet, waist circumference, BMI, plasma SFA, MUFA, PUFA and consumption of fruits and vegetables.

	Duration vegan diet	Waist circumference	BMI	SFA	MUFA	PUFA	Fruits and vegetables
	Vegans (n = 36)	(n = 72)	(n = 72)	(n = 72)	(n = 72)	(n = 72)	(n = 72)
Adiponectin [ng/ml]							
Model 1	−0.21 (0.21)	−0.40 (0.0005)	−0.26 (0.03)	−0.28 (0.02)	0.15 (0.20)	0.09 (0.45)	0.15 (0.21)
Model 2	−0.13 (0.48)	−0.34 (0.005)	−0.24 (0.06)	−0.26 (0.04)	0.08 (0.51)	0.16 (0.21)	0.17 (0.18)
hsCRP [mg/l]							
Model 1	−0.17 (0.32)	0.44 (0.0001)	0.39 (0.0007)	0.25 (0.03)	−0.04 (0.72)	−0.24 (0.04)	−0.08 (0.51)
Model 2	−0.35 (0.07)	0.37 (0.003)	0.35 (0.005)	0.12 (0.34)	0.07 (0.57)	−0.27 (0.03)	−0.03 (0.79)
ICAM-1 [ng/ml]							
Model 1	0.09 (0.59)	0.39 (0.0006)	0.24 (0.04)	0.12 (0.34)	−0.09 (0.43)	−0.03 (0.78)	−0.007 (0.95)
Model 2	0.11 (0.57)	0.31 (0.01)	0.16 (0.19)	0.04 (0.76)	−0.03 (0.81)	−0.05 (0.69)	−0.09 (0.48)
IL-18 [pg/ml]							
Model 1	0.35 (0.04)	0.04 (0.75)	0.04 (0.77)	0.11 (0.35)	−0.04 (0.74)	−0.07 (0.57)	−0.15 (0.20)
Model 2	0.44 (0.02)	−0.09 (0.49)	0.001 (0.99)	0.10 (0.42)	−0.10 (0.42)	−0.04 (0.78)	−0.07 (0.60)
IL-1 RA [pg/ml]							
Model 1	0.35 (0.03)	0.36 (0.0019)	0.24 (0.04)	0.10 (0.39)	−0.11 (0.38)	0.08 (0.50)	0.06 (0.60)
Model 2	0.34 (0.08)	0.42 (0.0006)	0.24 (0.06)	0.04 (0.75)	0.08 (0.55)	−0.03 (0.84)	0.003 (0.98)
Omentin-1 [ng/ml]							
Model 1	−0.30 (0.08)	−0.29 (0.01)	−0.27 (0.02)	−0.31 (0.008)	0.15 (0.20)	0.08 (0.51)	0.09 (0.44)
Model 2	−0.24 (0.21)	−0.26 (0.04)	−0.29 (0.02)	−0.30 (0.02)	0.08 (0.51)	0.16 (0.20)	−0.006 (0.96)
Resistin [ng/ml]							
Model 1	0.47 (0.004)	−0.13 (0.29)	−0.06 (0.63)	0.17 (0.15)	−0.18 (0.14)	0.07 (0.55)	−0.06 (0.64)
Model 2	0.59 (0.0008)	0.17 (0.19)	0.09 (0.49)	0.30 (0.02)	−0.07 (0.61)	−0.14 (0.27)	−0.15 (0.23)

Expressed as Spearman rho (p-value), Model 1: unadjusted, Model 2: mutually adjusted for age, sex, smoking status, education, waist circumference, physical activity, alcohol consumption.

## Discussion

To the best of our knowledge, the present cross-sectional study is the most comprehensive study, investigating a wide spectrum of inflammatory biomarkers in exclusive vegans compared to omnivores. However, we observed no significant differences of the inflammatory biomarkers hsCRP, adiponectin, ICAM-1, IL-18, IL-1 RA, omentin-1 or resistin between vegans and omnivores. Nevertheless, the duration of a vegan diet seemed to have an impact on impaired inflammatory profiles. Interestingly, we detected trends towards differences in inflammatory biomarkers dependent on plasma levels of SFA or PUFA. Further, the present study supports BMI and waist circumference as important influencing factors on the inflammatory state.

Scientific evidence has been suggested that systemic low-grade inflammation is linked to various diseases. Knowing that a vegan or vegetarian diet may be protective against many chronic diseases like type 2 diabetes^4^, cardiovascular diseases^5^, or cancer^6^, recent research hypothesized that plant-based nutritional habits might ameliorate inflammatory processes and accordingly, decrease circulating levels of inflammatory biomarkers and therefore might cause the reduced risk of chronic diseases in these populations^3,12–14^. However, up to date there is restricted evidence, limiting conclusions regarding the effect of a vegan diet on inflammatory biomarkers. Indeed, there are no studies, which perform comprehensive analyses to investigate the association of a vegan diet on the inflammatory profile. Accordingly, until now, only two studies provide data on inflammatory biomarkers in vegans (n = 9)^15^ or strict vegetarians (animal products less than once a month, n = 66)^16^ as small subgroups, while mainly investigating advanced glycation end products^15^ or gut microbiota composition^16^ in a vegetarian population. These studies showed conflicting results. Whereas Franco-de-Moraes *et al*.^16^ observed higher CRP levels in omnivores compared to vegans, Šebeková *et al*.^15^ noticed no differences in CRP levels. The latter is in line with our results. Nevertheless, these studies are mainly focused on CRP as one marker of inflammation, whereas the present study comprise a comprehensive investigation of a wide spectrum of inflammatory biomarkers in exclusive vegans compared to omnivores. Further, knowing that vegetarian patterns on inflammatory biomarkers have been investigated in more studies^3,14^, the limitations are similar. In fact, Craddock *et al*.^14^ recently mentioned in a systematic review and meta-analysis from 2019, that CRP was explored in seven studies, but significantly lowered concentrations following a vegetarian-based diet was observed in four studies only^14^. Further, the authors haven been noticed that except of CRP, other inflammatory and immune biomarkers of interest were not reported upon or were only explored in single studies, thereby limiting conclusions regarding the effect of vegetarian-based dietary patterns on the inflammatory profile^14^. Interestingly, Haghighatdoost *et al*.^3^ emphasize a trend towards lower CRP concentrations in subjects following vegetarian diet of at least 2 years, while no significant effect was found in participants with a duration time of less than 2 years, but at least 6 month^3^. In line with these observations, the present study noticed that ‘long-time’ vegans (>4.8 years) were more likely to have lower hsCRP level compared to vegans adhering to a vegan diet ≤ 4.8 years. Knowing, that scientific evidence proposed high resistin and IL-18 level to induce insulin resistance and promote inflammatory processes, thereby playing a central role in various metabolic, inflammatory and autoimmune diseases^17,18^, it was surprising, that increased resistin and IL-18 concentrations were highly correlated with the duration of a vegan diet, and in some degree, also IL-1 RA. This needs to be verified by further studies, as the effects of short-term or long-term adherence to a vegetarian or vegan diet on low-grade inflammatory state remain underestimated. Taken together, more research is highly warranted to evaluate associations between a vegan or vegetarian diet and inflammatory biomarkers to provide more evidence that a vegan or vegetarian diet may be beneficial to prevent or counteract inflammatory state and might be a nutritional approach to prevent risk of chronic diseases.

An array of nutrients as well as non-nutritive bioactive components of dietary habits may influence the inflammatory profile^14,19^. In this context, the type and quantity of dietary fat may be responsible for changes in inflammatory biomarkers^14,19^. Indeed, scientific evidence linked dietary SFA to an impaired inflammation profile^20^, while SFA intake is typically higher in non-vegetarians due to the consumption of animal products^2^. Vegetarian-based populations typically consume a greater proportion of unsaturated fatty acids than non-vegetarians^21^, proposed to be inversely associated with inflammation^22^. Interestingly, the present study observed lower plasma levels of SFA in vegans compared to omnivores, while the level of SFA was positive correlated to resistin. Inverse associations have been observed for the anti-inflammatory biomarker adiponectin and omentin-1. As expected, levels of PUFA were higher in vegans compared to omnivores, and corresponding higher plasma PUFA concentrations were inversely correlated with hsCRP concentration. Moreover, also intake of fruit and vegetables has been suggested to attenuation inflammation indicated by a large body of scientific evidence^23,24^. At the level of bioactive compounds occurring in fruits and vegetables, primarily carotenoids and flavonoids seem to modulate inflammatory as well as immunological processes^23,24^. Nevertheless, the present study detected no correlation between consumption of fruits and vegetables and inflammatory biomarkers.

It is important to note that overweight and obesity are associated with increased inflammation biomarkers^25^. In line, BMI and waist circumference were correlated with almost all investigated inflammatory biomarkers, even if obese individuals have been excluded from the study (exclusion criteria BMI ≥ 30 kg/m^2^). Nevertheless, these findings support higher BMI and increased waist circumference as important influencing factors for impaired inflammatory profiles although in non-obese participants.

The present study has several strengths. Our study covered a comprehensive spectrum of biomarkers that reflect inflammation, including a set of novel markers at the site of adipose-tissue induced inflammatory response. The study provides comprehensive high-quality data as a result of the standardized procedures, including the collection of blood, in combination with extensive information from computer-based questionnaires, dietary assessment by 3-day weighting protocol and anthropometric measurements, enabling us to adjust for the most important potential confounders. However, some limitations of our study deserve to be mentioned. First, the RBVD-study is relatively small, including 36 vegans and 36 omnivores. The study was powered for a primary research question about differences in bone health between vegans and omnivores and therefore it cannot be ruled out that some findings that were of borderline statistical significance may have been due to insufficient sample size. Second, the cross-sectional design of the present study does not allow for causal inference. Third, the study included middle-aged healthy German men and women, and therefore the results may not be generalized to other populations, such as other ethnic or age.

In conclusion, the RBVD-study is the first study providing a comprehensive spectrum of inflammatory biomarkers in vegans compared to omnivores, observing no significant differences of the inflammatory biomarkers hsCRP, adiponectin, ICAM-1, IL-18, IL-1 RA, omentin-1 or resistin between vegans and omnivores. Additional studies based on larger study populations are required to further evaluate the associations between a vegan diet and inflammatory biomarkers to provide more evidence that a vegan diet could provide means for prevention of chronic disease risk.

## Methods

### RBVD-Study

#### Study population

**Figure 1:**
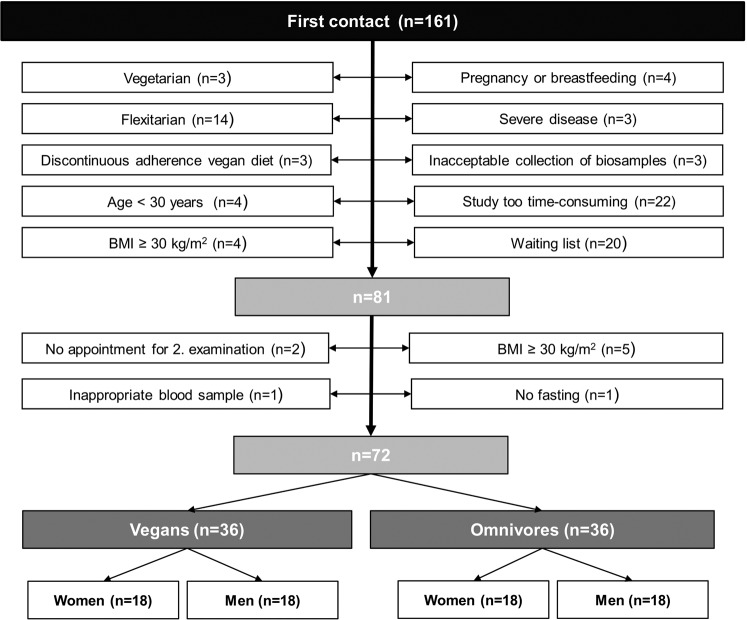
Flowchart.

Study participants were recruited by announcement and investigated from 01^st^ January 2017 until 31^th^ July 2017 in Berlin. Participants for the present study were individuals who responded to this advertisement, contacted the study center at the German Federal Institute of Risk Assessment (BfR) via phone or mail (n = 161), following by a phone screening consisted of a brief explanation of the study and checking inclusion criteria (age 30–60 years, following the diet at least 1 year) and exclusion criteria (BMI ≥ 30 kg/m^2^, cardiovascular disease, type 2 diabetes, cancer, pregnancy, breastfeeding, current infection). A omnivorous diet was defined as the consumption of at least three portions of meat per week or 2 portions of meat and 2 portions of processed meat per week, whereas a vegan diet was defined as no consumption of any animal food products. Each participant visited the study center twice. On their first visit, participants gave their written informed consent and received instructions to document their diet by a three-day weighed food protocol. At the second visit, anthropometric measurements and lifestyle characteristics were assessed, and a fasting blood sample was collected. As shown in Fig. 1, the final study population comprises 36 vegans and 36 omnivores, which were matched by sex- and age. The study was conducted in accordance with the Declaration of Helsinki. The study was approved by the Ethics Committee of Charité University Medical Center Berlin (No. EA4/121/16).

#### Assessment of lifestyle characteristics

Anthropometric measurements (weight, height, and waist circumference) were collected by trained and quality-monitored personnel, while participants wearing only light underwear and no shoes. Body weight was assessed by an electronic digital scale (Omron BF511, Germany) and the height was measured using a flexible anthropometer (SECA, Germany). The formula for BMI comprises weight in kilograms divided by height in meters squared. Waist circumference was defined as in the horizontal plane midway between the lowest ribs and the iliac crest. Information on physical activity, educational level and smoking status was assessed by computer-based questionnaires. The educational level was defined as ‘low education’ (no degree), ‘intermediate education’ (vocational school, technical college) or ‘high education’ (university, university of applied sciences). The amount of time spent on cycling, sports and gardening has been determined for summer and winter separately [hours/week]. Physical activity contains the sum of average hours in summer and winter per week of these activities. Walking comprises the sum of average hours per week during summer and winter. Dietary habits including alcohol consumption, fruit and vegetable consumption were assessed by three-day weighed food records for two week days and one weekend day. Data of the weighing protocols were merged with the German national food code (Bundeslebensmittelschlüssel Version 3.02, BLS) to assign with macro- and micronutrients. In this manuscript the fruit and vegetable consumption (g/d) includes the sum of the intake of fresh fruits, cooked, raw vegetables, as well as potatoes and legumes.

#### Blood collection and laboratory analysis

About 60 mL of venous blood was collected from fasting participants at the BfR study center. Several routine biomarkers including hsCRP were measured from fresh blood samples at the accredited medical analytics laboratory (Labor28 GmbH, Berlin, Germany) immediately at each study day. About half of blood was fractionated and aliquoted into serum, EDTA-plasma and erythrocytes, and stored in freezers (−80 °C) for conservation until time of analysis.

In 2018, inflammatory biomarkers other than hsCRP were measured in plasma samples at the Institute for Clinical Chemistry and Pathobiochemistry, Otto-von-Guericke University Magdeburg (Magdeburg, Germany). Plasma levels of IL-1 RA were analyzed using a sandwich ELISA from R&D Systems (Minneapolis, Minnesota, USA) with intra-assay coefficients of variation between 3.7% and 7.3%, inter-assay coefficients of variation between 6.7% and 11.0% and a limit of detection of 6.3 pg/ml. Plasma IL-18 was quantified with a sandwich ELISA by IBL (Gunma, Japan) with intra-assay coefficients of variation between 4.9% and 10.8%, inter-assay coefficients of variation between 5.2% and 10.0% and a limit of detection of 12.5 pg/ml. Plasma ICAM-1 concentrations were analyzed using an immunoassay from Life Technologies (Carlsbad, California, USA) with intra-assay coefficients of variation between 2.2% and 7.8%, inter-assay coefficients of variation between 5.1% and 11.1% and a limit of detection of 2.2 ng/ml. Serum concentrations of total adiponectin were also measured with a sandwich ELISA by ALPCO Diagnostics (Salem, New Hampshire, USA) with intra-assay coefficients of variation between 5.3% and 5.4%, inter-assay coefficient of variation between of 5.0% and a limit of detection of 0.019 ng/ml. Serum omentin-1 concentrations were measured with a sandwich ELISA by Biovendor (Brno, Czech Republic) with intra-assay coefficients of variation between 3.2% and 4.1%, inter-assay coefficients of variation between 4.4% and 4.8% and a limit of detection of 0.5 ng/ml. Plasma resistin levels were quantified with an sandwich ELISA from R&D Systems (Minneapolis, Minnesota, USA) with intra-assay coefficients of variation between 3.8% and 5.3%, inter-assay coefficients of variation between 7.8% and 9.2% and a limit of detection of 0.026 ng/ml. Fatty acids in plasma phospholipids were analyzed by gas chromatography by the German Institute of Human Nutrition in Potsdam-Rehbruecke^26^. The total saturated fatty acids (SFA), including myristic acid (C14:0), pentadecanoic acid (C15:0), palmitic acid (C16:0), heptadecanoic acid (C17:0), stearic acid (C18:0) and arachidic acid (C20:0). The total monounsaturated fatty acids (MUFA) containing palmitoleic acid (C16:1n7c), cis-vaccenic acid (C18:1n7c), oleic acid (C18:1n9c), as well as gondoic acid (C20:1n9). The total polyunsaturated fatty acids (PUFA) including alpha-linolenic acid (C18:3n3), eicosapentaenoic acid (C20:5n3), docosapentaenoic acid n-3 (C22:5n3) docosahexaenoic acid n-3 (C22:6n3), linoleic acid (C18:2n6c), gamma-linolenic acid (C18:3n6), docosadienoic acid (C20:2n6), dihomo-γ-linolenic acid (C20:3n6), arachidonic acid (C20:4n6), adrenic acid (C22:4n6), and docosapentaenoic acid n-6 (C22:5n6).

#### Sample size estimation

As the main research question of the RBVD-study is the investigation of bone health following a vegan diet compared to omnivores, the power calculation was based on the bone health measurement. The sample size was calculated, assuming a clinically relevant difference of at least 5% in bone health (estimated based on broadband ultrasound attenuation measurements) between vegan and omnivores. Along with a level of significance of 5% and a power of 80%, in total 72 participants were required (36 vegans, 36 omnivores) (G∗power, t test for independent samples).

#### Statistical analyses

Normally distributed variables were reported as mean and standard derivation (SD), skewed variables were reported as median and interquartile range (IQR) and log-transformed for analyses. Categorical variables were reported as percentage. For comparison of the characteristics of vegans compared to omnivores, a Chi-Square test for categorical variables and a Student’s t test or Mann–Whitney U test for continuous variables were used.

To investigate the association of veganism with inflammatory biomarkers compared to omnivores, an analysis of variance (ANOVA) was performed for model 1 (unadjusted). Additionally, a multivariable adjusted analysis of covariance (ANCOVA) was conducted to detected differences between vegans and omnivores in model 2, adjusted for age, sex, smoking status, education, waist circumference, physical activity and alcohol consumption. Independent of the type of diet, differences in inflammatory biomarkers according to the duration of a vegan diet, waist circumference, BMI, total SFA, total MUFA, total PUFA and fruit and vegetable consumption, have been investigated. Correlations between inflammatory biomarkers and duration of a vegan diet (in vegans only, n = 36), waist circumference, BMI, total SFA, total MUFA, total PUFA and fruit/vegetable consumption were assessed using spearman correlation (unadjusted, Model 1) and spearman partial correlation mutually adjusted for age, sex, smoking status, education, waist circumference, physical activity, alcohol consumption (model 2). For model 2, a multivariable adjusted ANCOVA were performed to investigate differences in inflammatory biomarkers according to the duration of a vegan diet (cut off median: 4.8 years). Skewed variables that were log-transformed before ANOVA or ANOVA were back-transformed and expressed as geometric means and 95%-CI.

Sensitivity analyses were carried out after exclusion of participants taking anti-rheumatic or analgesic drugs. In detail, analyses were performed after exclusion of participants with regular intake of anti-rheumatic and analgesic drugs (at least 3 times within last week before examination) (n = 2), additionally participants with single dose within the last week before examination (n = 7), participants with single dose within in the last two weeks before examination (n = 18), participants with single dose within the last month before examination (n = 27). Further, in sensitivity analyses extreme concentrations of the inflammatory biomarkers have been excluded (≤1^st^ or ≥ 99^th^ percentile), respectively (n = 2). To eliminate the influence of the type of diet i.e. a vegan or omnivorous diet, the spearman partial correlations have been additionally adjusted for type of diet. All statistical analyses were performed using SAS software, version 9.4 (SAS institute, Cary, N.C., USA). P values of<0.05 were considered statistically significant.

## Data Availability

The datasets generated during and/or analyzed during the current RBVD-Study are not publicly available due to provisions of the written informed consent.

## References

[1] Allès Benjamin, Baudry Julia, Méjean Caroline, Touvier Mathilde, Péneau Sandrine, Hercberg Serge, Kesse-Guyot Emmanuelle (2017). Comparison of Sociodemographic and Nutritional Characteristics between Self-Reported Vegetarians, Vegans, and Meat-Eaters from the NutriNet-Santé Study. Nutrients.

[2] Craig WJ (2010). Nutrition concerns and health effects of vegetarian diets. Nutr Clin Pract.

[3] Haghighatdoost F, Bellissimo N, Totosy de Zepetnek JO, Rouhani MH (2017). Association of vegetarian diet with inflammatory biomarkers: a systematic review and meta-analysis of observational studies. Public Health Nutr.

[4] Lee, Y. & Park, K. Adherence to a Vegetarian Diet and Diabetes Risk: A Systematic Review and Meta-Analysis of Observational Studies. *Nutrients* **9**, 10.3390/nu9060603 (2017). 10.3390/nu9060603 PMC5490582 28613258

[5] Kahleova H, Levin S, Barnard ND (2018). Vegetarian Dietary Patterns and Cardiovascular Disease. Prog Cardiovasc Dis.

[6] Dinu M, Abbate R, Gensini GF, Casini A, Sofi F (2017). Vegetarian, vegan diets and multiple health outcomes: A systematic review with meta-analysis of observational studies. Crit Rev Food Sci Nutr.

[7] Liu C (2016). Adiponectin, TNF-alpha and inflammatory cytokines and risk of type 2 diabetes: A systematic review and meta-analysis. Cytokine.

[8] Jefferis BJ (2011). Interleukin 18 and coronary heart disease: prospective study and systematic review. Atherosclerosis.

[9] Pearson TA (2003). Markers of inflammation and cardiovascular disease: application to clinical and public health practice: A statement for healthcare professionals from the Centers for Disease Control and Prevention and the American Heart Association. Circulation.

[10] Shibata R, Ouchi N, Murohara T (2009). Adiponectin and cardiovascular disease. Circ J.

[11] Li S, Shin HJ, Ding EL, van Dam RM (2009). Adiponectin levels and risk of type 2 diabetes: a systematic review and meta-analysis. JAMA.

[12] Eichelmann F, Schwingshackl L, Fedirko V, Aleksandrova K (2016). Effect of plant-based diets on obesity-related inflammatory profiles: a systematic review and meta-analysis of intervention trials. Obes Rev.

[13] Barbaresko J, Koch M, Schulze MB, Nothlings U (2013). Dietary pattern analysis and biomarkers of low-grade inflammation: a systematic literature review. Nutr Rev.

[14] Craddock Joel C, Neale Elizabeth P, Peoples Gregory E, Probst Yasmine C (2019). Vegetarian-Based Dietary Patterns and their Relation with Inflammatory and Immune Biomarkers: A Systematic Review and Meta-Analysis. Advances in Nutrition.

[15] Sebekova K (2001). Plasma levels of advanced glycation end products in healthy, long-term vegetarians and subjects on a western mixed diet. Eur J Nutr.

[16] Franco-de-Moraes AC (2017). Worse inflammatory profile in omnivores than in vegetarians associates with the gut microbiota composition. Diabetol Metab Syndr.

[17] Acquarone E, Monacelli F, Borghi R, Nencioni A, Odetti P (2019). Resistin: A reappraisal. Mech Ageing Dev.

[18] Dinarello CA, Novick D, Kim S, Kaplanski G (2013). Interleukin-18 and IL-18 binding protein. Front Immunol.

[19] Galland L (2010). Diet and inflammation. Nutr Clin Pract.

[20] Ruiz-Nunez B, Dijck-Brouwer DA, Muskiet FA (2016). The relation of saturated fatty acids with low-grade inflammation and cardiovascular disease. J Nutr Biochem.

[21] Davis BC, Kris-Etherton PM (2003). Achieving optimal essential fatty acid status in vegetarians: current knowledge and practical implications. Am J Clin Nutr.

[22] Kalogeropoulos N (2010). Unsaturated fatty acids are inversely associated and n-6/n-3 ratios are positively related to inflammation and coagulation markers in plasma of apparently healthy adults. Clin Chim Acta.

[23] Watzl B (2008). Anti-inflammatory effects of plant-based foods and of their constituents. Int J Vitam Nutr Res.

[24] Lapuente, Estruch, Shahbaz, Casas (2019). Relation of Fruits and Vegetables with Major Cardiometabolic Risk Factors, Markers of Oxidation, and Inflammation. Nutrients.

[25] Ellulu MS, Patimah I, Khaza’ai H, Rahmat A, Abed Y (2017). Obesity and inflammation: the linking mechanism and the complications. Arch Med Sci.

[26] Weitkunat K (2017). Odd-chain fatty acids as a biomarker for dietary fiber intake: a novel pathway for endogenous production from propionate. Am J Clin Nutr.

